# Evaluating two live-attenuated vaccines against *Salmonella enterica* serovar Reading in turkeys: reduced tissue colonization and cecal tonsil transcriptome responses

**DOI:** 10.3389/fvets.2024.1502303

**Published:** 2024-12-19

**Authors:** Melissa S. Monson, Manoj Gurung, Bradley L. Bearson, Samuel J. Whelan, Julian M. Trachsel, Torey Looft, Matthew J. Sylte, Shawn M.D. Bearson

**Affiliations:** ^1^Food Safety and Enteric Pathogens Research Unit, National Animal Disease Center, U.S. Department of Agriculture-Agricultural Research Service (USDA-ARS), Ames, IA, United States; ^2^Oak Ridge Institute for Science and Education, Agricultural Research Service (ARS) Research Participation Program, Oak Ridge, TN, United States; ^3^Agroecosystems Management Research Unit, National Laboratory for Agriculture and the Environment, U.S. Department of Agriculture-Agricultural Research Service (USDA-ARS), Ames, IA, United States

**Keywords:** *Salmonella*, Reading, turkey, vaccine, cross-protection, colonization, cecal tonsil, transcriptome

## Abstract

Vaccines that cross-protect across serovars of *Salmonella enterica* (*Salmonella*) would be a beneficial intervention against emerging and persistent *Salmonella* isolates of concern for the turkey industry. The 2017–2019 foodborne outbreak of *Salmonella enterica* serovar Reading (*S*. Reading) revealed the need for effective control of this serovar in turkey production. This study evaluated two live-attenuated *Salmonella* vaccines, an internally developed cross-protective vaccine and a commercially available vaccine, against an outbreak-associated strain of *S*. Reading in turkeys. At 1 day and 3 weeks of age, male turkey poults were either mock-vaccinated with phosphate buffered saline (PBS) or given one of the vaccines by oral gavage (primary and booster) or aerosol spray (primary) then drinking water (booster). At 7 weeks of age, poults were challenged with 10^9^ colony forming units (CFU) of *S*. Reading; a mock-vaccinated group was mock-challenged with PBS. Colonization of the cecal contents and cecal tonsil was 1.5–3 log_10_ CFU/g lower in vaccinated birds than mock-vaccinated birds at 7 and/or 14 days post-inoculation (DPI). *Salmonella* dissemination to the spleen was significantly reduced by both vaccines. Gene expression of intestinal transporters (such as *SCNN1B* and *SLC10A2*) and tight junction proteins was significantly decreased in the turkey cecal tonsil transcriptome at 2 DPI with *S*. Reading. Vaccination with either vaccine mitigated most cecal tonsil gene expression responses to *S*. Reading challenge. Therefore, both the internally developed vaccine and commercial vaccine were cross-protective against colonization and dissemination, and both were able to limit transcriptional changes from challenge in intestinal health-related genes in the cecal tonsil, thereby providing vaccination efficacy and impact data against *S*. Reading in turkeys.

## 1 Introduction

*Salmonella enterica* subspecies *enterica* is a human foodborne pathogen responsible for an estimated 1.3 million food-related illnesses in the U.S. annually (1). Food producing animals are frequently colonized with *Salmonella* sub-clinically and can unknowingly contaminate animal production environments and processing facilities, resulting in *Salmonella*-tainted food products. The Centers for Disease Control and Prevention (CDC) and other government agencies have implicated poultry products in various foodborne outbreaks (2). In an outbreak from November 2017 to March 2019, *Salmonella enterica* serovar Reading (*S*. Reading) linked to turkeys and turkey products resulted in 358 human infections and one death across 42 U.S. states and Canada (3, 4). As neither a single turkey product nor a common supplier of turkey products was identified as the source of the outbreak, it has been proposed that the rapid and uniform introduction and transmission of this emergent *S*. Reading strain occurred vertically in commercial turkey production (5, 6). Furthermore, because of its impact on food safety, *Salmonella* is frequently listed on the top ten list of health and production issues for the turkey industry (7). Thus, the turkey industry would benefit from broadly protective intervention strategies to control emerging and persistent *Salmonella* isolates of concern to reduce the burden of human illness and industry costs due to product recalls.

Currently, vaccination against *Salmonella* is the primary pre-harvest intervention administered in turkey production, frequently involving a commercial vaccine in breeder flocks and autogenous vaccines employed in commercial production. With numerous serovars of *Salmonella* isolated in the various phases of turkey production and processing, a potential limitation with current vaccination strategies is the lack of cross-protection against the >2,600 serovars of *Salmonella*. *Salmonella* evades cross-protection by expressing serovar-specific carbohydrate and protein antigens on the bacterial outer surface that are immunodominant, thereby inducing a host immune response that is specific to that *Salmonella* serovar. To improve *Salmonella* vaccinology for food producing animals, our research team created a live-attenuated vaccine that limits *Salmonella* serovar-specific immunity to promote non-serovar-specific responses (8). Turkeys vaccinated with this internally developed vaccine showed a significant reduction in intestinal and systemic colonization following challenge with multidrug-resistant *Salmonella enterica* serovar Heidelberg from the 2011 ground turkey outbreak (9). Evidence also supports cross-protection across serovars using the only commercial vaccine approved for use against *Salmonella* in turkeys in the U.S. (10, 11). In the current study, we evaluated our internally developed, patented, cross-protective *Salmonella* vaccine BBS 866 along with the commercial AviPro^®^ Megan^®^ Egg vaccine (Elanco US Inc., Greenfield, IN, USA) to assess reduction of *S*. Reading isolate SX 446 (FSIS21923883) in turkeys (12). Vaccination with BBS 866 or AviPro^®^ Megan^®^ Egg significantly reduced colonization and dissemination of *S*. Reading in turkeys, and vaccination mitigated transcriptional changes in intestinal health-related genes induced by *S*. Reading in turkey cecal tonsil. Therefore, both vaccines are cross-protective interventions to minimize *S*. Reading in turkeys.

## 2 Materials and methods

### 2.1 Ethics statement

All procedures in this study involving animals were approved by and conducted according to the regulations established by the National Animal Disease Center Institutional Animal Care and Use Committee.

### 2.2 Bacterial strains and selective media

This experiment evaluated two live-attenuated *Salmonella* vaccines (Table 1), the commercial AviPro^®^ Megan^®^ Egg vaccine (Elanco US Inc.) and an internally developed cross-protective BBS 866 DIVA (differentiation of infected from vaccinated animals) vaccine (8, 9). In the culture media described below, antibiotics were used at the following concentrations: nalidixic acid, 30 μg/mL; ampicillin, 100 μg/mL; streptomycin, 100 μg/mL; novobiocin, 40 μg/mL; and kanamycin, 50 μg/mL. To prepare for primary and booster vaccinations, the BBS 866 vaccine strain was grown statically at 37°C in LB (Lennox) broth (Thermo Fisher Scientific Inc., Waltham, MA, USA) with nalidixic acid for ~19 h. Resulting cultures were pelleted, resuspended in sterile phosphate buffered saline (PBS) (Sigma-Aldrich Co., St. Louis, MO, USA) with 15% glycerol, quantified by plating on LB agar (Sigma-Aldrich Co.), and stored at −80°C until use. Prior to primary and booster vaccinations, a vial (2,500 doses) of the AviPro^®^ Megan^®^ Egg vaccine was resuspended according to manufacturer's instructions and diluted using sterile PBS. The dose of each vaccine administered via both vaccination delivery protocols was confirmed by quantitative bacteriology on LB agar.

**Table 1:** *Salmonella enterica* strains.

	**BBS 866**	**AviPro Megan Egg**	**SX 446 (FSIS21923883)**
Purpose	Vaccine	Vaccine	Challenge
Serovar	Typhimurium	Typhimurium	Reading
Background strain	χ4232 wildtype	SR-11 wildtype	wildtype
Genotype	Δ*rybB* Δ*omrAB* Δ*micA* Δ*invRrfaH*::*neogyrA*^C248T^	*Δcya Δcrp*	*aph(3″)-Ib aph(6)-Id bla_*TEM*−1_ sul2 tet(A)*
Phenotype^a^	Nal, Km		Str, Amo, Amp, Sul, Tet

^a^Antibiotics abbreviated as Nal, nalidixic acid; Km, kanamycin; Str, streptomycin; Amo, amoxicillin; Amp, ampicillin; Sul, sulfamethoxazole; Tet, tetracycline.

A turkey *Salmonella enterica* serovar Reading strain SX 446 (FSIS21923883) with multidrug resistance (i.e., streptomycin, sulfamethoxazole, tetracycline, amoxicillin, ampicillin) was used for the challenge (Table 1). To prepare the inoculum, the strain was grown in LB broth with ampicillin and streptomycin in a stationary culture at 37°C for ~21 h. The *S*. Reading culture was pelleted, resuspended in sterile PBS with 15% glycerol, quantified by plating on LB agar, and stored at −80°C. The inoculum dose [targeted 1 × 10^9^ colony forming units (CFU)] was verified by quantitative bacteriology on LB agar after challenge.

Qualitative enrichments for *Salmonella* were performed using tetrathionate broth (VWR International LLC, Radnor, PA, USA) followed by Rappaport–Vassiliadis medium (Becton, Dickinson & Company, Franklin Lakes, NJ, USA) as previously described (13). Xylose lysine tergitol-4 (XLT-4) agar (Becton, Dickinson & Company) with 4.6 μL/mL tergitol (0.46% final volume) and brilliant green agar with sulfadiazine (BGS) (Becton, Dickinson & Company) were used to confirm that poults were enrichment negative for *Salmonella* at arrival. XLT-4 agar without antibiotics was also used to confirm the mock-challenged group remained negative throughout the trial. XLT-4 agar supplemented with novobiocin was employed to detect the AviPro^®^ Megan^®^ Egg vaccine the day prior to *S*. Reading challenge, while recovery of the BBS 866 vaccine on the day before challenge was performed using XLT-4 agar supplemented with nalidixic acid and kanamycin. After challenge, growth media for assessing prevalence and bacterial load of *S*. Reading in turkey samples was XLT-4 agar supplemented with streptomycin and ampicillin.

### 2.3 Animal trial and bacteriology

**Figure 1:**
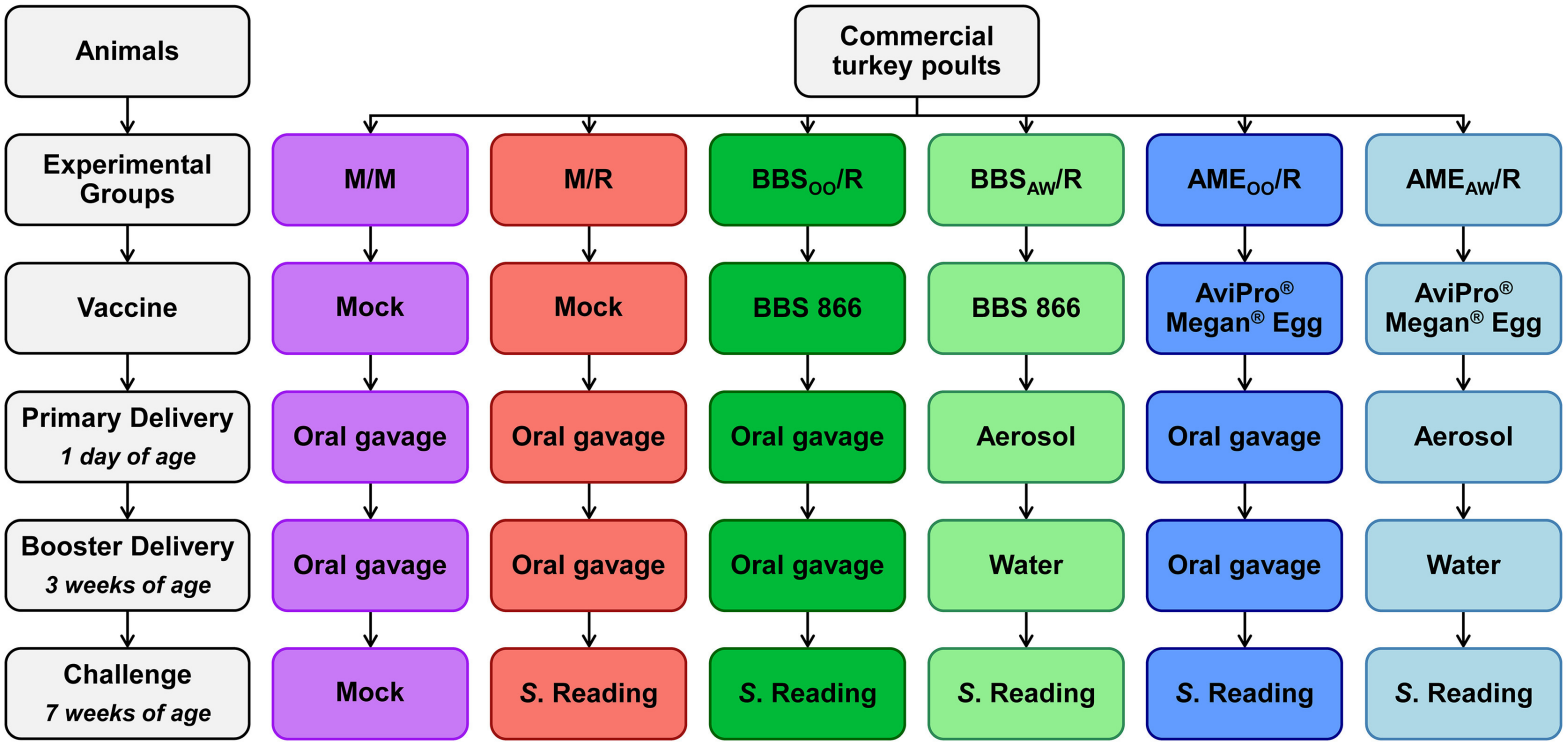
Experimental design for vaccination and challenge of each group of turkeys. At 1-day and 3-weeks of age, poults in four groups were administered PBS (M/M and M/R), BBS 866 (BBS_OO_/R), or AviPro^®^ Megan^®^ Egg (AME_OO_/R) via oral gavage (*n* = 64 birds/group). The two remaining groups received BBS 866 (BBS_AW_/R), or AviPro^®^ Megan^®^ Egg (AME_AW_/R) via aerosol and water booster (*n* = 40 birds/group). At 7-weeks of age, poults were orally inoculated with PBS (M/M) or *S*. Reading (all other vaccination/challenge groups).

Six groups were examined in this study (Figure 1): mock-vaccinated/mock-challenged (M/M), mock-vaccinated/*S*. Reading-challenged (M/R), BBS 866-vaccinated via oral gavage/*S*. Reading-challenged (BBS_OO_/R), BBS 866-vaccinated via aerosol and water/*S*. Reading-challenged (BBS_AW_/R), AviPro^®^ Megan^®^ Egg-vaccinated via oral gavage/*S*. Reading-challenged (AME_OO_/R), or AviPro^®^ Megan^®^ Egg-vaccinated via aerosol and water/*S*. Reading-challenged (AME_AW_/R). One-day-old male Nicholas turkey poults were obtained from a commercial hatchery and randomly divided into six ABSL-2 isolation rooms, each room housing one vaccination/challenge group. Poults were given *ad libitum* access to water and a game bird starter ration (Purina Animal Nutrition LLC, Arden Hills, MN, USA) throughout the entire study. A random subset of poults (*n* = 5 birds) was euthanized upon arrival and qualitative bacteriology by enrichment was performed on the intestinal tract as previously described (13); *Salmonella* was not detected.

After placement in the rooms, primary vaccinations were performed via oral gavage or aerosol spray. The mock-vaccinated groups (M/M and M/R) received 0.25 mL of sterile PBS by oral gavage (*n* = 64 birds/group). The BBS_OO_/R and AME_OO_/R groups (*n* = 64 birds/group) received 5 × 10^8^ CFU of BBS 866 or AviPro^®^ Megan^®^ Egg vaccine by oral gavage (0.25 mL at 2 × 10^9^ CFU/mL of vaccine). Turkeys in the BBS_AW_/R and AME_AW_/R groups (*n* = 40 birds/group) each received ~5 × 10^8^ CFU of BBS 866 or AviPro^®^ Megan^®^ Egg vaccine via 1 mL applied by a fine mist from a handheld spray bottle. At 3 weeks of age, booster vaccinations of the poults were performed by oral gavage or provided through drinking water. The M/M and M/R groups received 0.5 mL of sterile PBS by oral gavage. The BBS_OO_/R and AME_OO_/R groups received 4 × 10^8^ CFU of BBS 866 or AviPro^®^ Megan^®^ Egg vaccine by oral gavage (0.5 mL at 8 × 10^8^ CFU/mL of vaccine). To booster vaccinate the BBS_AW_/R and AME_AW_/R groups, the drinking water in each room was replaced with water containing 1.2 × 10^8^ CFU/mL of BBS 866 or AviPro^®^ Megan^®^ Egg vaccine for ~2 h (target dose of 4–5 × 10^8^ CFU). The dose for both vaccines was based on the quantity of commercial vaccine provided for 2,500 doses/vial, which was consistent with a previous study using the BBS 866 vaccine in turkeys (9).

Necropsies were performed at −1, 2, 7, 14, and 21 days post-inoculation (DPI). Poults were randomly selected from each group for necropsy and euthanized using sodium pentobarbital at label dose. At 7 weeks of age, poults from the M/M (*n* = 4 birds) and M/R (*n* = 8 birds) groups were necropsied 1 day prior to challenge (−1 DPI) to confirm mock-vaccinated poults remained *Salmonella* negative. Additionally, at −1 DPI, the BBS_OO_/R (*n* = 12 birds) and AME_OO_/R (*n* = 10 birds) groups were sampled to detect persistence of the vaccines. On the next day, poults in the five *S*. Reading-challenged groups were given 1 × 10^9^ CFU of *S*. Reading (SX 446) by oral gavage (0.5 mL at 2 × 10^9^ CFU/mL of inoculum). The M/M group received 0.5 mL of sterile PBS by oral gavage. At 2 DPI, the M/M, M/R, BBS_OO_/R, and AME_OO_/R groups were sampled (*n* = 12 birds/group) for transcriptomics (see below) with bacteriology performed to determine bacterial load. The BBS_AW_/R and AME_AW_/R groups were not sampled at −1 DPI or 2 DPI as these groups were used to assess vaccination by aerosol and water delivery in comparison to oral gavage. Twelve birds/group were sampled from all six vaccination/challenge groups at 7 and 14 DPI and all remaining birds (*n* = 12–16 birds/group) were sampled at 21 DPI. Samples of neck skin (2 and 7 DPI only), cloaca, spleen, cecal contents, and cecal tonsil were aseptically collected from each bird and kept on ice until processed; the neck skin, cloaca, and cecal tonsil were separately and aseptically rinsed with sterile PBS to remove non-adherent bacteria from the tissue surfaces. Cecal tonsil samples from the M/M, M/R, BBS_OO_/R, and AME_OO_/R groups were collected into 10 mL of RNAlater (Thermo Fisher Scientific Inc.) at 2 DPI, perfused at 4°C for 24 h, and then stored at −80°C until use.

To detect and enumerate *S*. Reading at selected time points following inoculation, 1 gram (g) or the maximum available (if < 1 g) of tissue sample (neck skin, cloaca, spleen, or cecal tonsil) or cecal contents was used for quantitative and qualitative bacteriology as previously described (13). Briefly, each tissue was homogenized using a Stomacher 80 Biomaster (Seward Inc., Bohemia, NY, USA) with 2 mL sterile PBS/g of tissue as the diluent. One hundred microliters of the homogenized tissues and 10-fold serial dilutions were plated on selective media and incubated for 48 h at 37°C to obtain quantitative counts. *Salmonella* positive colonies were counted for each sample and the *Salmonella* CFU/g of tissue was calculated. If < 1 g of tissue or cecal contents was used for bacteriology, resulting colony counts were adjusted proportionally to be equivalent to the count in 1 g. Two step enrichments were also performed from the homogenized tissues and streaked onto selective media to qualitatively detect *Salmonella*. Samples that were positive for *Salmonella* only by enrichment were randomly assigned an integer between 1 and 19 CFU/g (20 CFU/g = limit of detection), while samples negative for *Salmonella* by both plating and enrichment were assigned 0 CFU/g. For each tissue, the enumeration data was log_10_ transformed and statistically analyzed using a non-parametric Kruskal-Wallis rank sum test (significance threshold = *p*-values < 0.05). For each tissue with a significant effect of vaccination on colonization, pairwise comparisons between vaccinations and delivery protocols were performed using a Dunn's test [significance threshold = false discovery rate-adjusted *p*-values (FDR) < 0.05].

### 2.4 RNA isolation and sequencing

RNA-sequencing (RNA-seq) was performed to understand acute responses to *S*. Reading and the effect of vaccination on the gene expression in turkey cecal tonsil. Total RNA was extracted from cecal tonsil tissues (sampled from one cecal tonsil as other was used for bacteriology above) collected at 2 DPI from the M/M, M/R, BBS_OO_/R, and AME_OO_/R groups (*n* = 12 birds/group) using the RNAqueous Total RNA Isolation kit (Thermo Fisher Scientific Inc.) with manufacturer's protocols modified to incorporate an on-column DNase treatment (RNase-free DNase Set; QIAGEN Sciences, Germantown, MD, USA). RNA quality and quantity (RIN scores ≥ 9.1) were determined using a 2100 Bioanalyzer (Agilent Technologies Inc., Santa Clara, CA, USA) before submission to the Iowa State University DNA Facility for library preparation and sequencing. Stranded mRNA-seq cDNA libraries were constructed using the NEBNext Ultra II Directional RNA Library Prep kit (New England Biolabs, Ipswich, MA, USA) with the NEBNext Multiplex Oligos for Illumina (96 Unique Dual Index Primer Pairs) kit (New England Biolabs) for sample indexing. Libraries were pooled and sequenced on a NovaSeq 6000 (Illumina Inc., San Diego, CA, USA) using 4 lanes (*n* = 4 technical replicates/sample) of an S4 flow cell to produce 100-cycle paired-end reads.

### 2.5 Bioinformatic analyses and differential expression testing

Raw reads were processed using Trimmomatic v.0.39 (14) to filter and trim (adapters removed with Illuminaclip, end trimmed if quality score < 20, and entire reads removed if average quality score < 30 or minimum length < 75 bp). Filtered reads were mapped to the reference genome *Meleagris gallopavo* 5.1 (GCF_000146605.3) using STAR v.2.7.10b (15) with default parameters. Resulting BAM files were sorted by name with samtools v.1.17 (16) and gene expression quantified using HTSeq v.2.0.2 (17) with default parameters (except stranded = reverse) to count reads that uniquely mapped to genes (NCBI Annotation Release 103) across the turkey genome.

Differential expression analysis was completed using DESeq2 v.1.34.0 (18). Technical replicates were collapsed into one set of read counts per sample and used to normalize for library size, estimate dispersions, and fit a negative binomial GLM (design = ~ isolation batch + vaccination/challenge group). Design of the GLM was determined based on likelihood ratio tests (FDR < 0.05). To understand the distribution of the datasets, variance stabilized read counts were used to calculate sample-to-sample distances and to perform principal component analysis (PCA) on the 500 genes with highest between-sample variance. Pairwise Wald tests were run on normalized read counts using the GLM design above to determine genes with significant differential expression between each *S*. Reading-challenged group and the M/M group (significance thresholds = |log_2_ fold changes (log_2_FC)| ≥ 1.0 and FDR < 0.05).

In order to fully interpret the differential expression in the turkey, unannotated genes with significant differential expression were BLAST aligned (Megablast with default parameters; E-value < 1e-50, percent identity ≥ 90%; https://blast.ncbi.nlm.nih.gov) to all chicken and turkey genes in the NCBI Nucleotide (NT) database (https://www.ncbi.nlm.nih.gov/nuccore) to identify additional gene names. Many genes in the turkey genome are incompletely annotated as locus (LOC) numbers yet have named orthologs in chicken that can be extrapolated to the turkey genes. Annotating to chicken also revealed that nearly 20% of the significantly differentially expressed turkey genes might be gene fragments instead of paralogs, due to chicken gene sequences that were split into more than one non-overlapping gene in the turkey genome annotation. For downstream analysis, suspected gene fragments were assigned to the same shared gene name but kept as separate differentially expressed genes. Using both gene names from the turkey annotation and those identified by chicken orthologs, GO-Slim Biological Process terms were associated to the significantly differentially expressed genes using the chicken GO term database in PANTHER v.19.0 (19). An over-representation test based on a Fisher's Exact test with FDR correction (significance threshold = FDR < 0.05) was performed for each comparison to M/M to predict the functional impacts of the significant differential expression.

## 3 Results and discussion

### 3.1 Persistence of the vaccine strains in orally vaccinated turkeys

Two live-attenuated *Salmonella* vaccine strains, BBS 866 and AviPro^®^ Megan^®^ Egg, both derived from *Salmonella enterica* serovar Typhimurium, were utilized to vaccinate poults in this study. At 7 weeks of age (prior to *S*. Reading challenge; 4 weeks post-booster vaccination), both vaccine strains were recoverable by qualitative bacteriology from a subset of cecal contents (up to 50%), cecal tonsils (up to 40%), and cloaca (up to 25%) collected from the BBS_OO_/R and AME_OO_/R groups (Table 2). Neither vaccine strain was detected in the spleen. A previous study vaccinated 3-week-old turkeys with a single dose of BBS 866 and observed the vaccine strain in the cecum and cloaca for 2 weeks, but did not detect the vaccine in the spleen, nor the crop, liver, or bursa of Fabricius (9). In this study, both vaccine strains were able to persist within the intestinal tract of a subset of poults for at least 4 weeks after vaccination. Vaccine-origin isolates [similar to AviPro^®^ Megan^®^ Vac 1 (Elanco US Inc.) or AviPro^®^ Megan^®^ Egg (Elanco US Inc.)] have been observed in chicken and turkey samples obtained by the Food Safety and Inspection Service (FSIS) and the Food and Drug Administration (FDA) (20), indicating persistence can occur during the production cycle.

**Table 2:** Both *Salmonella* vaccine strains were recoverable from the turkey intestine prior to challenge at 7 weeks of age^a^.

**Tissue**	**Vaccine^b, c^**	**Prevalence**	
		**Positive/total**	**Percent of total**
Cecal contents	BBS_OO_	6/12	50%
	AME_OO_	0/10	0%
Cecal tonsil	BBS_OO_	3/12	25%
	AME_OO_	4/10	40%
Cloaca	BBS_OO_	3/12	25%
	AME_OO_	1/10	10%
Spleen	BBS_OO_	0/12	0%
	AME_OO_	0/10	0%

^a^Samples collected 1 day prior to challenge; 4 weeks after booster vaccination.

^b^BBS_OO_, BBS 866-vaccinated via oral gavage; AME_OO_, AviPro^®^ Megan^®^ Egg-vaccinated via oral gavage.

^c^Groups vaccinated by aerosol and water (BBS_AW_ and AME_AW_) were not sampled at −1 DPI.

### 3.2 Impact of vaccination on tissue colonization by *Salmonella* Reading in turkeys

**Figure 2:**
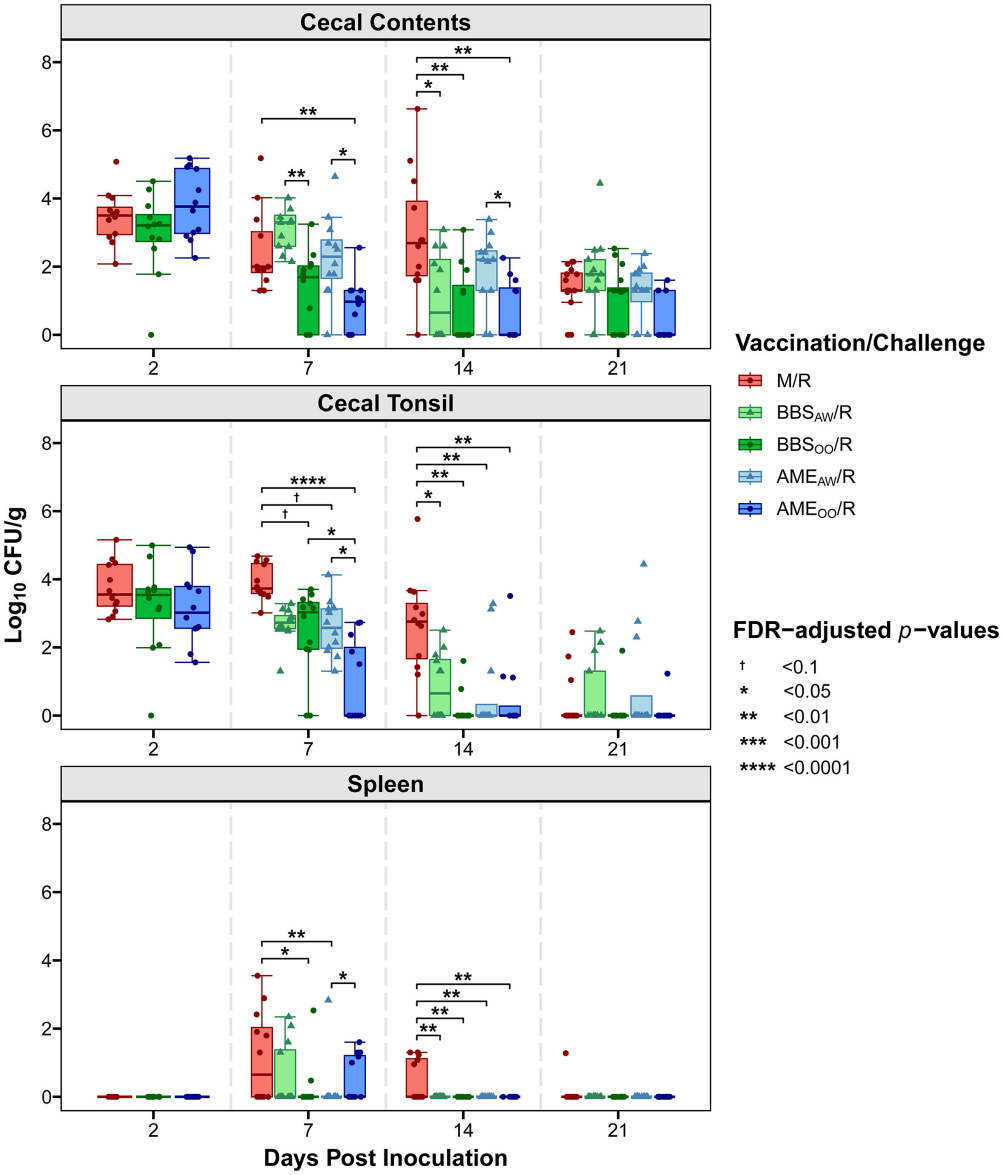
Vaccination reduced colonization and dissemination of *S*. Reading in turkeys. At 1-day and 3-weeks of age, poults were administered PBS (M/R), BBS 866 via oral gavage (BBS_OO_/R), BBS 866 via aerosol and water (BBS_AW_/R), AviPro^®^ Megan^®^ Egg via oral gavage (AME_OO_/R), or AviPro^®^ Megan^®^ Egg via aerosol and water (AME_AW_/R). Poults were inoculated with *S*. Reading at 7-weeks of age. Colony forming units (CFU) of *Salmonella* were measured in a gram of tissue (or cecal contents) collected at 2, 7, 14, or 21 days post-inoculation (DPI) (*n* = 12–16/group) and are shown log_10_ transformed. Statistical significance was assessed with pairwise Dunn's tests (FDR < 0.05) within cecal contents, cecal tonsil or spleen. Colonization of cloaca is shown in Supplementary Figure 1.

**Figure 3:**
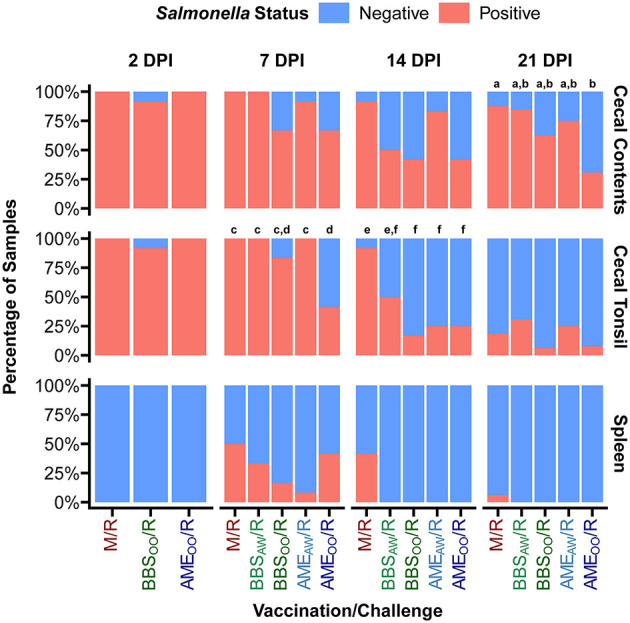
Prevalence of *S*. Reading positive samples in the turkeys. At 1-day and 3-weeks of age, poults were administered PBS (M/R), BBS 866 by oral gavage (BBS_OO_/R), BBS 866 by aerosol and water (BBS_AW_/R), AviPro^®^ Megan^®^ Egg by oral gavage (AME_OO_/R), or AviPro^®^ Megan^®^ Egg by aerosol and water (AME_AW_/R) prior to challenge with *S*. Reading at 7-weeks of age. Cecal contents and tissues were collected at 2, 7, 14, and 21 days post-inoculation (DPI) and *Salmonella* prevalence determined by quantitative and qualitative bacteriology. Letters indicate vaccination/challenge groups (*n* = 12–16/group) with significantly different (FDR < 0.05) proportions of *Salmonella* positive samples based on pairwise Fisher's tests performed within each tissue and DPI. Prevalence in cloaca is shown in Supplementary Figure 1.

A primary goal of this study was to evaluate our *Salmonella* vaccine BBS 866 and the AviPro^®^ Megan^®^ Egg vaccine against a multidrug-resistant *S*. Reading strain obtained by FSIS from comminuted turkey in 2019 (12). All birds in the M/M group remained *Salmonella* negative throughout the study. Tissue colonization by *S*. Reading was measured at 2, 7, 14, and 21 DPI (Figure 2). At 2 DPI, no significant differences in *Salmonella* load were observed between the *S*. Reading-challenged groups, with ~3–4 log_10_ CFU/g in cecal contents and cecal tonsil, and no dissemination to the spleen. Initial *Salmonella* colonization of the cloaca was lower than the intestinal samples and no significant differences between groups were observed in the cloaca at any DPI (Supplementary Figure 1). Only four neck skin samples were *Salmonella* positive at 2 DPI (2 in M/R and 2 in BBS_OO_/R) and none of the skin samples were positive at 7 DPI; therefore, neck skin was not an appreciable site of *Salmonella* colonization in this study and was not assessed at 14 or 21 DPI. At 7 DPI, the birds in the AME_OO_/R group had a significant 3 log_10_ CFU/g reduction in cecal tonsil colonization compared to the M/R group (Figure 2). The AME_OO_/R group also had 1.6 log_10_ CFU/g lower *Salmonella* load in the cecal contents at 7 DPI. When compared to the M/R group, dissemination to the spleen at 7 DPI was significantly reduced in the BBS_OO_/R and AME_AW_/R groups. Vaccination had a more consistent impact on colonization at 14 DPI, significantly reducing *Salmonella* load by 1.8–2.4 log_10_ CFU/g in cecal tonsil for all four vaccinated groups and by 1.8–2.2 log_10_ CFU/g in cecal contents in three vaccinated groups (excluding AME_AW_/R). At 14 DPI, *S*. Reading was still present at a low level (average 0.49 log_10_ CFU/g) in ~40% of spleens from the M/R group but was no longer detectable in any of the four vaccinated groups (Figures 2, 3). No significant differences in bacterial load were observed at 21 DPI in any tissue (Figure 2). However, the proportion of *Salmonella* positive cecal contents was significantly lower in the AME_OO_/R group compared to M/R at 21 DPI (Figure 3). Compared to M/R, prevalence was also significantly lower in cecal tonsil for AME_OO_/R at 7 DPI and for three vaccinated groups (excluding BBS_AW_/R) at 14 DPI.

The current experiment also assessed the impact of vaccination delivery on *Salmonella* load, comparing vaccination via aerosol spray and water booster, as in commercial production, to vaccination by oral gavage. Birds vaccinated by oral gavage had significantly lower *Salmonella* load at 7 DPI in cecal contents (both BBS_OO_/R and AME_OO_/R) and in cecal tonsil (just AME_OO_/R) compared to aerosol and water vaccinated groups (Figure 2). Conversely, *Salmonella* dissemination to spleen was significantly higher in AME_OO_/R than AME_AW_/R at 7 DPI. The AME_OO_/R group had significantly less colonization of cecal contents than the AME_AW_/R group at 14 DPI, with no significant differences in cecal tonsil and no detection in any vaccinated group in spleen after 7 DPI. Oral gavage delivery was performed because we expected to have greater consistency in vaccine dosage when directly inoculating a fixed volume into the crop. However, despite the inter-bird variability inherent in internalizing *Salmonella* from the aerosol spray or from consuming the booster dose in the drinking water, vaccination by aerosol and water booster (BBS_AW_/R and AME_AW_/R) successfully reduced both colonization and dissemination of *S*. Reading in turkeys.

Although reports of *Salmonella* vaccine trials in turkeys are limited, both vaccines examined herein have previously shown cross-protection against at least one serovar of *Salmonella* in turkeys. Vaccination of turkeys by oral gavage with BBS 866 prior to challenge was shown to significantly reduce both colonization (cecum) and dissemination (spleen) by multidrug-resistant *Salmonella enterica* serovar Heidelberg (9). The commercial vaccine, when given to turkeys by aerosol and water booster, decreased colonization of *Salmonella enterica* serovars Infantis and Hadar in cecum and cecal tonsil (and cloaca for *S*. Infantis) (10). Dissemination of *S*. Infantis was reduced in spleen and not observed in bone marrow when turkeys were vaccinated prior to challenge. *S*. Hadar had minimal to no dissemination (only two positive spleen samples) in the experiment. Previous research has also shown that vaccinating turkeys with the commercial vaccine can significantly reduce overall *Salmonella* load in the cecum when poults were subsequently challenged with a mixture of *Salmonella enterica* serovars Reading, Agona and Saintpaul (11). Despite providing two booster doses via water (at 3 weeks and again at 5 weeks), based on *Salmonella* prevalence, vaccination was not sufficient to decrease dissemination to the spleen after challenge with their mixed culture. Although this previous study included *S*. Reading as part of the challenge, they only examined the impacts of vaccination on *Salmonella* collectively and did not differentiate between the three serovars in the mixed culture. In the current study, the effect of both vaccines was evaluated specifically against *S*. Reading. *Salmonella* prevalence in intestinal samples from birds given either vaccine was significantly decreased. Furthermore, both colonization (cecal samples) and dissemination (spleen) of *Salmonella* were quantitatively decreased by the commercial vaccine at 7 and 14 DPI. *Salmonella* load was also significantly lower in poults vaccinated with BBS 866 with significant reduction in dissemination to spleen at 7 and 14 DPI and most of the intestinal samples reduced at 14 DPI. Therefore, both vaccines were cross-protective against *S*. Reading in turkeys.

### 3.3 Transcriptomic responses to *Salmonella* Reading and the effect of vaccination in turkey cecal tonsil

To assess the acute host responses to *S*. Reading colonization, the cecal tonsil transcriptome was investigated at 2 DPI with *S*. Reading or PBS (as mock-challenge). Approximately 85% of the resulting reads mapped uniquely to the turkey genome annotation, which revealed a total of 17,778 expressed genes in the turkey cecal tonsil. Although principal component analysis (Supplementary Figure 2) showed similar within-group and between-group variability, nearly half of the *S*. Reading-challenged samples (primarily from the M/R and AME_OO_/R groups) had large sample-to-sample distances (Supplementary Figure 3) from a portion of the M/M samples. The large variation in gene expression patterns make it appear as though challenge with *S*. Reading had limited effects on cecal tonsil expression, yet the M/M group had detectable differences from the M/R and AME_OO_/R groups.

Gene expression in three *S*. Reading-challenged groups (M/R, AME_OO_/R, BBS_OO_/R) was compared to the M/M. In total, 615 genes had significant differential expression (|log_2_FC| ≥ 1.0, FDR < 0.05) in these comparisons; 48% of these differentially expressed genes (297) had annotated gene names in the turkey genome, and the rest were assigned to a LOC number (Supplementary Table 1). To more fully capture the functions of these genes, unannotated differentially expressed genes were BLAST aligned to all chicken and turkey genes in the NCBI NT database. An additional 179 differentially expressed genes (56% of significant LOC genes) were attributed to a gene annotation based on their chicken ortholog.

**Figure 4:**
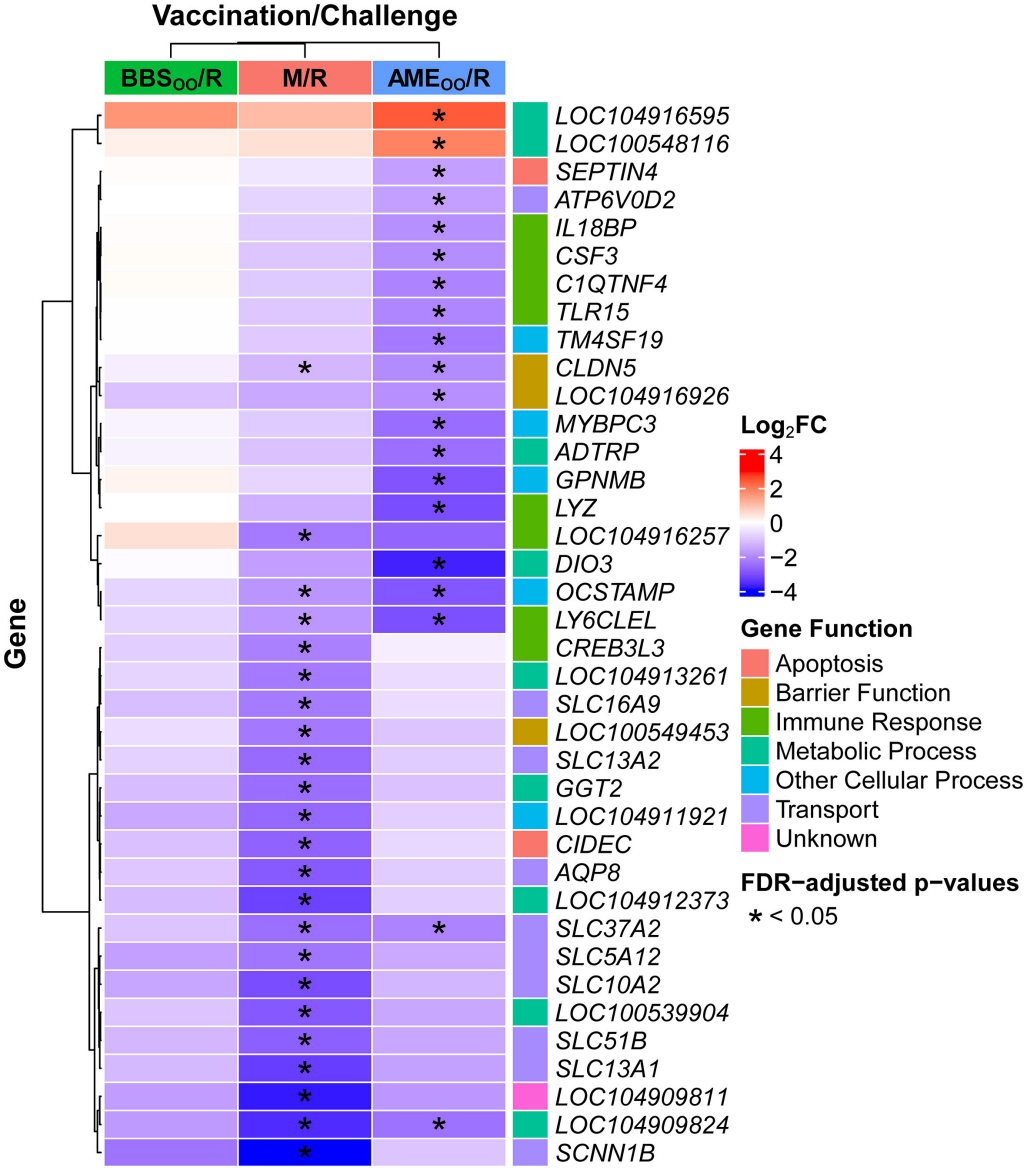
Top 20 differentially expressed genes in cecal tonsil for each *S*. Reading challenged group compared to the M/M group at 2 DPI. Significantly differentially expressed genes (|log_2_FC| ≥ 1.0 and FDR < 0.05) were determined using DESeq2. Based on magnitude of |log_2_FC|, the top 20 significantly differentially expressed genes for each comparison to the M/M group are shown (no significantly differentially expressed genes were observed between BBS_OO_/R and M/M), resulting in a total of 38 genes (due to overlapping genes in the top 20 for M/M and for AME_OO_/R). Asterisks indicate in which comparison(s) the gene has significant differential expression. M/M, mock-vaccinated/mock-challenged; M/R, mock-vaccinated/*S*. Reading-challenged; AME_OO_/R, AviPro^®^ Megan^®^ Egg-vaccinated via oral gavage*/S*. Reading-challenged; BBS_OO_/R, BBS 866-vaccinated via oral gavage/*S*. Reading-challenged; DPI, days post-inoculation; FC, fold change; FDR, Benjamini Hochberg adjusted *p*-values.

Most of the significantly differentially expressed genes occurred in the M/R group, of which 36 genes had increased expression and 542 were decreased compared to the M/M group (Supplementary Table 1). Despite the role of the cecal tonsil as a major gut-associated lymphoid tissue (GALT) in birds, only a few genes with significant differential expression after *S*. Reading challenge at 7 weeks of age were immune-related, including decreases in a S100A9-like gene (*LOC104916257*), lymphocyte antigen 6E-like (*LY6CLEL*), and cAMP responsive element binding protein 3 like 3 (*CREB3L3*) (Figure 4; Supplementary Table 1). Research in chickens has observed an acute (< 4 DPI) pro-inflammatory response to *Salmonella*, followed by an immunosuppressive shift to tolerance that allows for persistent colonization of the cecum (21–23). Conversely, inflammatory gene expression was not broadly induced at 2 DPI in turkey cecal tonsil despite colonization of this tissue by up to 4 logs of *S*. Reading in this study. Only five immune-related genes slightly increased in M/R compared to M/M (all log_2_FC < 2.0), including C-X-C motif chemokine ligand 13-like 3 (*CXCL13L3*), MHCB C-type lectin-like 1 (*BLEC1*), interleukin 6 (*IL6*), and two gene segments that are part of the T cell receptor alpha locus (Supplementary Table 1). Transcriptional responses during the acute phase (2 DPI) have been shown to vary dramatically by serovar in turkey blood leukocytes, with almost no differential expression after *S*. Heidelberg challenge, while *S*. Typhimurium induced a strong immune-related response (24, 25). Further research is needed in turkeys to establish what host or pathogen factors influence the initial response and whether pro-inflammatory responses, when they occur, also rapidly shift to tolerance as seen in chickens.

**Figure 5:**
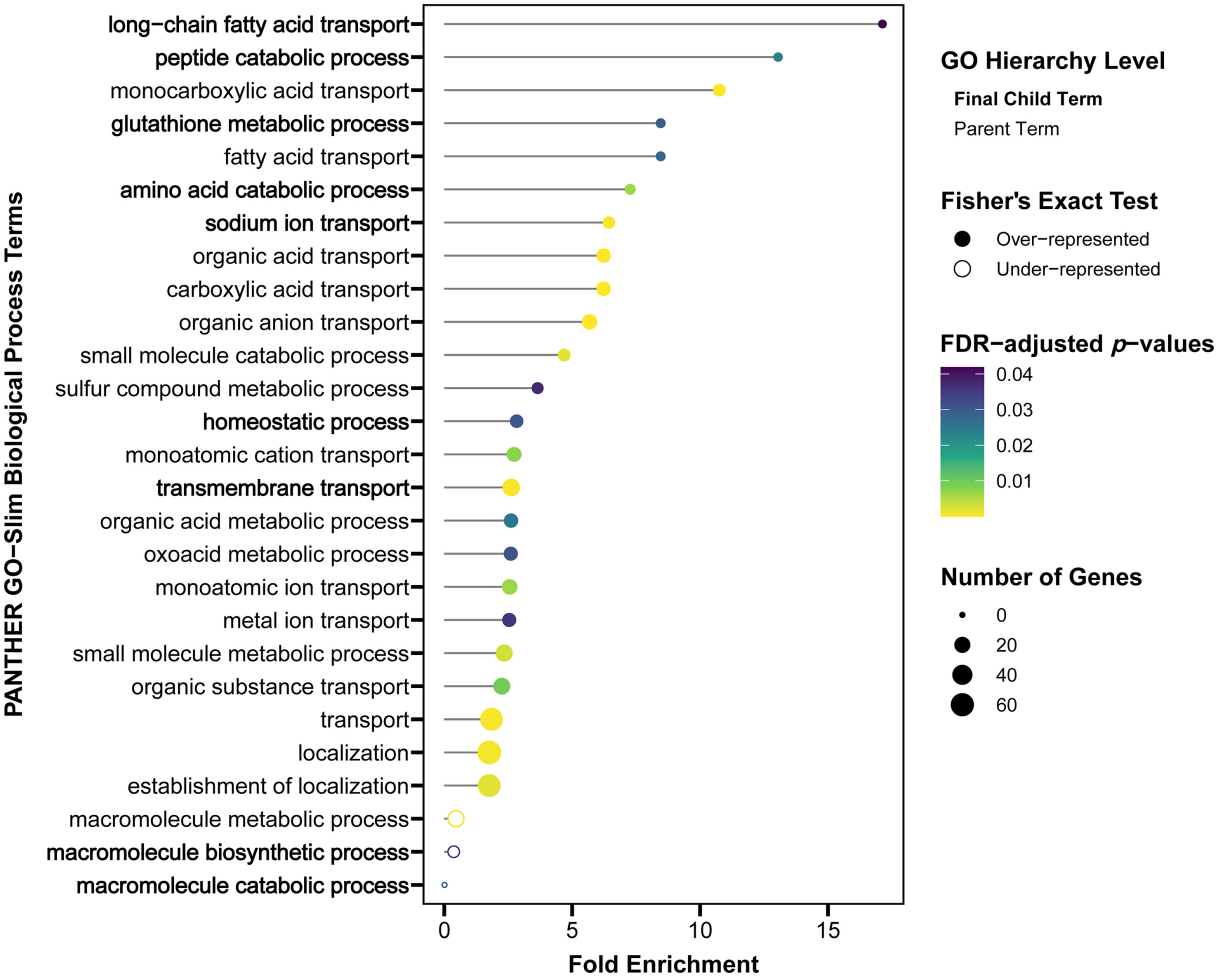
Transport and metabolic GO-Slim Biological Process terms were over-represented by the differentially expressed genes in the cecal tonsil of the M/R group compared to the M/M group at 2 DPI. Significantly over- or under-represented GO terms were based on a Fisher's Exact test and fold enrichments are shown, with significance indicated by color and the number of differentially expressed genes linked to the GO term represented by size. The most specific GO terms (final children of parent terms) are listed on the *y*-axis in bold. M/R, mock-vaccinated/*S*. Reading-challenged; M/M, mock-vaccinated/mock-challenged; DPI, days post-inoculation; GO, gene ontology.

After *S*. Reading challenge, only six genes had highly decreased expression (log_2_FC ≤ −3.0), of which three were transporters (Figure 4), sodium channel epithelial 1 beta subunit (*SCNN1B*), solute carrier family 10 member 2 (*SLC10A2*) and solute carrier family 14 member 1 (*SLC13A1*). Nine of the top 20 significantly differentially expressed genes in M/R compared to M/M were directly associated with cellular transport functions (Figure 4; Supplementary Table 1). Within the PANTHER database, 25 GO terms were over-represented by the differentially expressed genes, highlighting metabolic-related functions and transport terms like “long-chain fatty acid transport,” “sodium ion transport,” and “transmembrane transport” (Figure 5; Supplementary Table 2). In Awad et al. (26), *Salmonella enterica* serovar Enteritidis reduced ion transport in chicken intestinal tissues *ex vivo*, which is consistent with the reduced *SCNN1B* expression observed in turkey cecal tonsil in this study. *SCNN1B* encodes a subunit of the epithelial sodium channel ENaC that transports sodium through the apical epithelial membrane from the lumen and regulates fluid balance in the intestine (27). *SLC10A2* (*ASBT*) is an apical sodium-dependent bile acid transporter shown to have decreased expression in the ileum of *S*. Typhimurium challenged pigs (28). Other members of the FXR pathway of bile acid absorption, fatty acid binding proteins 2 and 6 (*FABP2* and *FABP6*) and nuclear receptor subfamily 1 group H member 4 (*NR1H4*), were also reduced in this swine study. *FABP2* and *FABP6* are fatty acid transporters also observed to be reduced by *Salmonella* in chickens (29–31). After *S*. Reading challenge in the current study, *SLC10A2, FABP2, FABP6*, and *NR1H4* decreased in turkey cecal tonsil, and as predicted in swine, could reflect compromised bile acid absorption when *Salmonella* is introduced into the intestine. *S*. Reading colonization also reduced expression of nutrient transporters like solute carrier family 5 member 1 (*SLC5A1*) and solute carrier family 15 member 1 (*SLC15A1*), which encode the glucose transporter SGLT1 and the amino acid transporter PEPT1, respectively. Reduced expression of *SLC5A1* or *SLC15A1* has been previously observed in chickens challenged with *S*. Enteritidis, *Campylobacter jejuni* or *Eimeria praecox* (32–34). In total, over 40 intestinal transporters from more than 20 gene families had reduced expression in turkey cecal tonsil, suggesting that intestinal uptake of nutrients, absorption of bile acids, and modulation of ion balance may be inhibited in the acute response to *S*. Reading. Further studies would be needed to investigate whether colonization by *S*. Reading causes detectable signs of pathology due to disruption of these intestinal functions in the turkey cecal tonsil.

Whether *Salmonella* colonization increases or decreases intestinal permeability in poultry has been inconsistent in previous studies. One study using *ex vivo* intestinal tissues observed that *S*. Enteritidis reduced permeability by electrophysical conductance measures (26). However, other research suggests that *Salmonella* negatively impacts the integrity of the intestinal barrier, which increases permeability and facilitates paracellular transport of the bacteria through the tight junctions between intestinal epithelial cells (35–37). In broilers, challenge with *S*. Typhimurium reduces gene expression of claudin 1 (*CLDN1*) and occludin (*OCLN*) in ileum (35) and *CLDN1*, claudin 4 (*CLDN4*) and *OCLN* in jejunum (36). Both studies also observed signs of intestinal injury due to *Salmonella* colonization, such as reduced villus height/crypt depth ratios. In the current study, challenge with *S*. Reading significantly decreased expression of seven tight junction-related genes, including gap junction protein beta 1 (*GJB1*), a gap junction alpha-3 protein-like gene (*LOC100551358*), claudin 5 (*CLDN5*), tight junction protein 3 (*TJP3*; also known as *ZO-3*) and *OCLN* (Figure 4; Supplementary Table 1). These genes have a positive role in the formation and maintenance of epithelial tight junctions and their decrease could facilitate *S*. Reading colonization of turkey intestinal tissues, like the cecal tonsil, and subsequent systemic dissemination.

Vaccination prior to *S*. Reading challenge mitigated most of the expression changes induced by *Salmonella* in the M/R group. No genes had significant differential expression in the BBS_OO_/R group compared to the M/M group. In the AME_OO_/R group, 9 genes were significantly increased and 39 decreased in comparison to the M/M group, of which three genes had a log_2_FC ≤ −3.0 (iodothyronine deiodinase 3 (*DIO3*), lysozyme (*LYZ*), and *LY6CLEL*) (Figure 4; Supplementary Table 1). Beyond *LYZ* and *LY6CLEL*, a few additional immune-related cytokines, receptors, and regulatory genes had significantly reduced expression in turkey cecal tonsil after challenge in the AME_OO_/R group (Supplementary Table 1), but no GO-Slim terms were over-represented in the AME_OO_/R group. Only 11 differentially expressed genes were shared between the M/R and AME_OO_/R comparisons to M/M, including decreased expression of an amine sulfotransferase-like gene (*LOC104909824*), *LY6CLEL*, osteoclast stimulatory transmembrane protein (*OCSTAMP*), solute carrier family 37 member 2 (*SLC37A2*) and *CLDN5*. Overall, the majority of differential expression in the M/R group was not observed if the birds were vaccinated prior to the challenge. Only *CLDN5*, a tight-junction related gene, and two solute transporters remained significantly lower in the AME_OO_/R group; this could be indicative of a decreased impact of *Salmonella* on intestinal health and barrier functions and may contribute to the lower colonization of *S*. Reading observed in the vaccinated birds (Figure 2).

## 4 Conclusions

Reducing *Salmonella* load and prevalence through cross-protective vaccination strategies would benefit the turkey industry and could reduce the risk of human foodborne illnesses. This study evaluated two live-attenuated vaccines against subsequent challenge with an outbreak-associated strain of *S*. Reading. Both the internally developed and the commercial vaccine were cross-protective and able to reduce colonization (cecal contents and cecal tonsil) and dissemination (spleen) of *S*. Reading in turkeys. *S*. Reading challenge also decreased gene expression of intestinal transporters and tight junction proteins in turkey cecal tonsil at 2 DPI. Reduced expression of these genes could reflect an acute disruption of intestinal health and barrier functions, which could play a role in allowing colonization by *S*. Reading. Most of the expression changes seen in cecal tonsil of unvaccinated birds were not observed in the poults vaccinated prior to *S*. Reading challenge, suggesting that vaccination may improve the bird's ability to maintain normal intestinal functions and impede colonization of *S*. Reading. Both vaccines in this study were cross-protective against *S*. Reading and could be used as interventions against colonization of this serovar in turkeys.

## Data Availability

The mRNA-seq datasets generated for this study can be found in the NCBI SRA database under BioProject accession number: PRJNA1189541. Raw read counts generated through analysis of the datasets are also available in the NCBI GEO database under accession number: GSE283163. All scripts used for analysis and figure generation are available at https://github.com/msmonson/SReading_CT_RNA-seq.git. *Salmonella* colonization data are available from the corresponding author upon reasonable request.
