# Supplementary material for: Evaluating two live-attenuated vaccines against Salmonella enterica serovar Reading in turkeys: reduced tissue colonization and cecal tonsil transcriptome responses
Source: Front Vet Sci. 2024 Dec 19;11:1502303. doi: 10.3389/fvets.2024.1502303 (PMC11694450; doi:10.3389/fvets.2024.1502303)
Supplement: Supplementary file 1 [file Image_1.pdf]

(A)

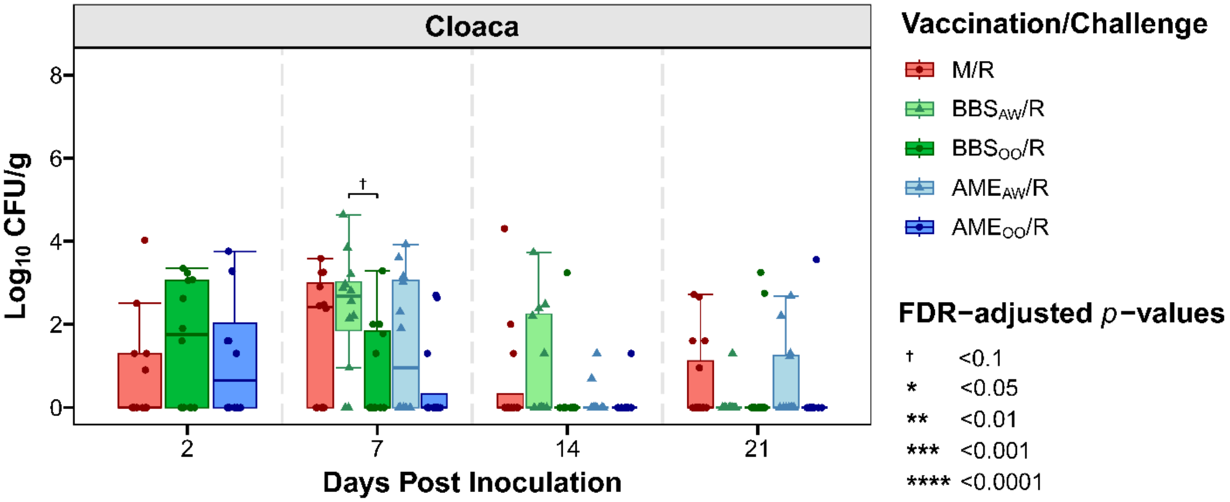

(B)

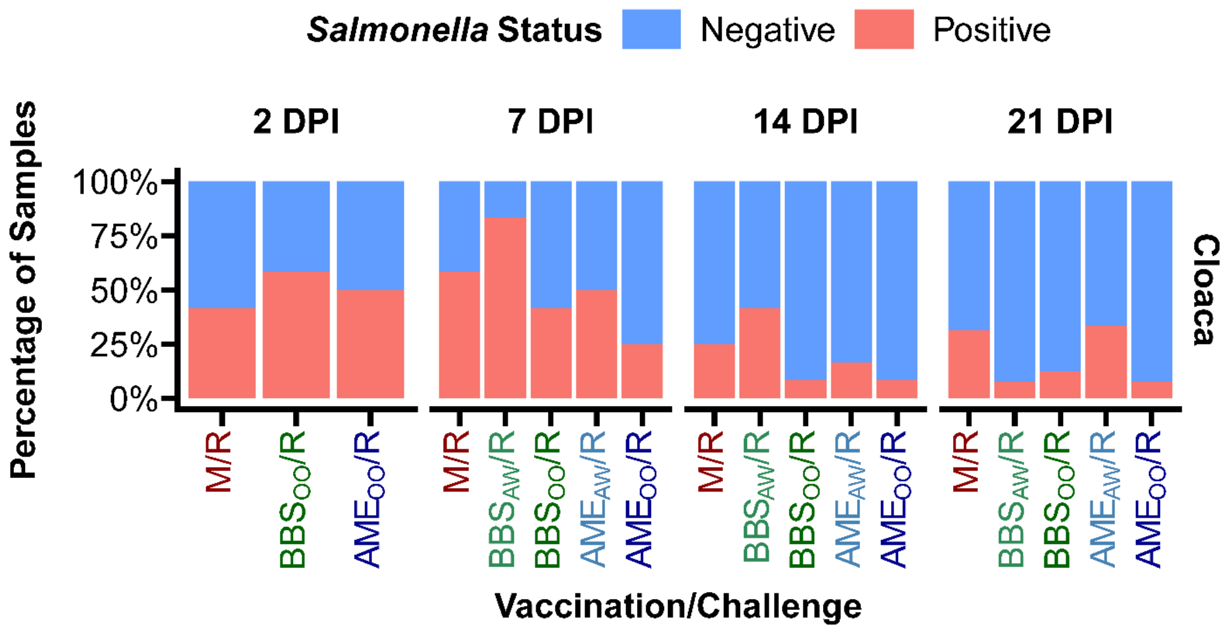

Supplementary Figure 1. Colonization and prevalence of *S*. Reading in turkey cloaca were not impacted by vaccination. At 1-day and 3-weeks of age, poults were administered PBS (M/R), BBS 866 by oral gavage (BBS_{OO}/R), BBS 866 by aerosol and water (BBS_{AW}/R), AviPro® Megan® Egg by oral gavage (AME_{OO}/R), or AviPro® Megan® Egg by aerosol and water (AME_{AW}/R). Poults were inoculated at 7-weeks of age with *S*. Reading (SX 446). **(A)** Colonization by *S*. Reading. Colony forming units (CFU) of *Salmonella* were measured in a gram of tissue (n = 12-16/group) collected at 2, 7, 14, or 21 days post-inoculation (DPI) and are shown $\log_{10}$ transformed. Statistical significance was assessed with pairwise Dunn's tests (FDR < 0.05). **(B)** Prevalence of *S*. Reading. Proportion of *Salmonella* positive samples in each group (n = 12-16/group) at 2-, 7-, 14-, or 21-DPI were determined by quantitative and qualitative bacteriology and compared using pairwise Fisher's Exact tests.
