# Supplementary material for: Evaluating two live-attenuated vaccines against Salmonella enterica serovar Reading in turkeys: reduced tissue colonization and cecal tonsil transcriptome responses
Source: Front Vet Sci. 2024 Dec 19;11:1502303. doi: 10.3389/fvets.2024.1502303 (PMC11694450; doi:10.3389/fvets.2024.1502303)
Supplement: Supplementary file 2 [file Image_2.pdf]

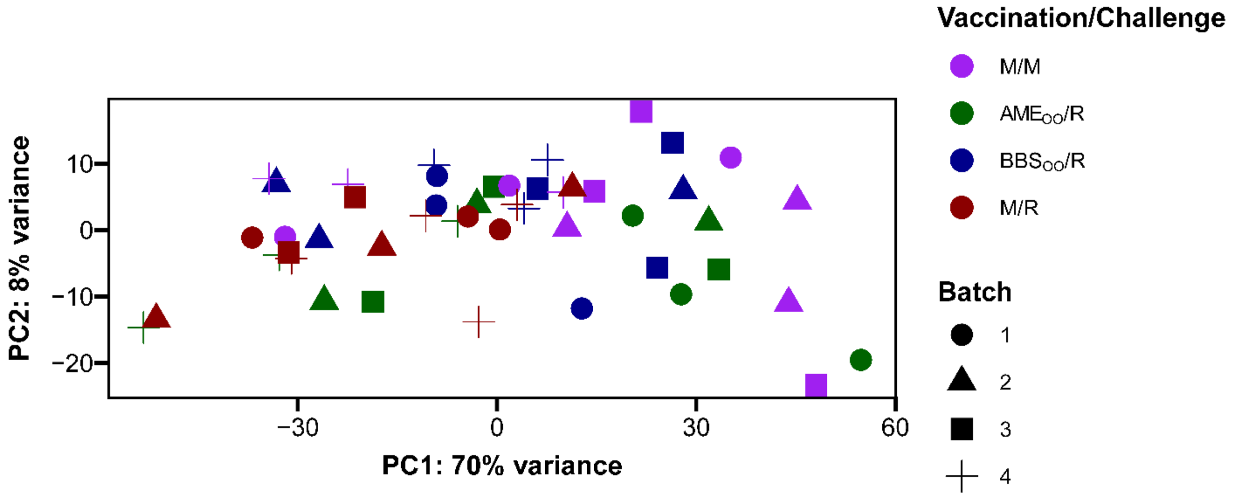

Supplementary Figure 2. Cecal tonsil transcriptomic datasets from 2 DPI variable outside of vaccination/challenge group and isolation batch. Principal components (PCs) were calculated using variance stabilized read counts for the 500 most variable genes. Datasets are shown according to group (color) and isolation batch (shape). M/M = mock-vaccinated/mock-challenged; M/R = mock-vaccinated/*S*. Reading-challenged; AME$_{OO}$/R = AviPro® Megan® Egg-vaccinated via oral gavage/*S*. Reading-challenged; BBS$_{OO}$/R = BBS 866-vaccinated via oral gavage/*S*. Reading-challenged; DPI = days post-inoculation.
