# Supplementary material for: Evaluating two live-attenuated vaccines against Salmonella enterica serovar Reading in turkeys: reduced tissue colonization and cecal tonsil transcriptome responses
Source: Front Vet Sci. 2024 Dec 19;11:1502303. doi: 10.3389/fvets.2024.1502303 (PMC11694450; doi:10.3389/fvets.2024.1502303)
Supplement: Supplementary file 3 [file Image_3.pdf]

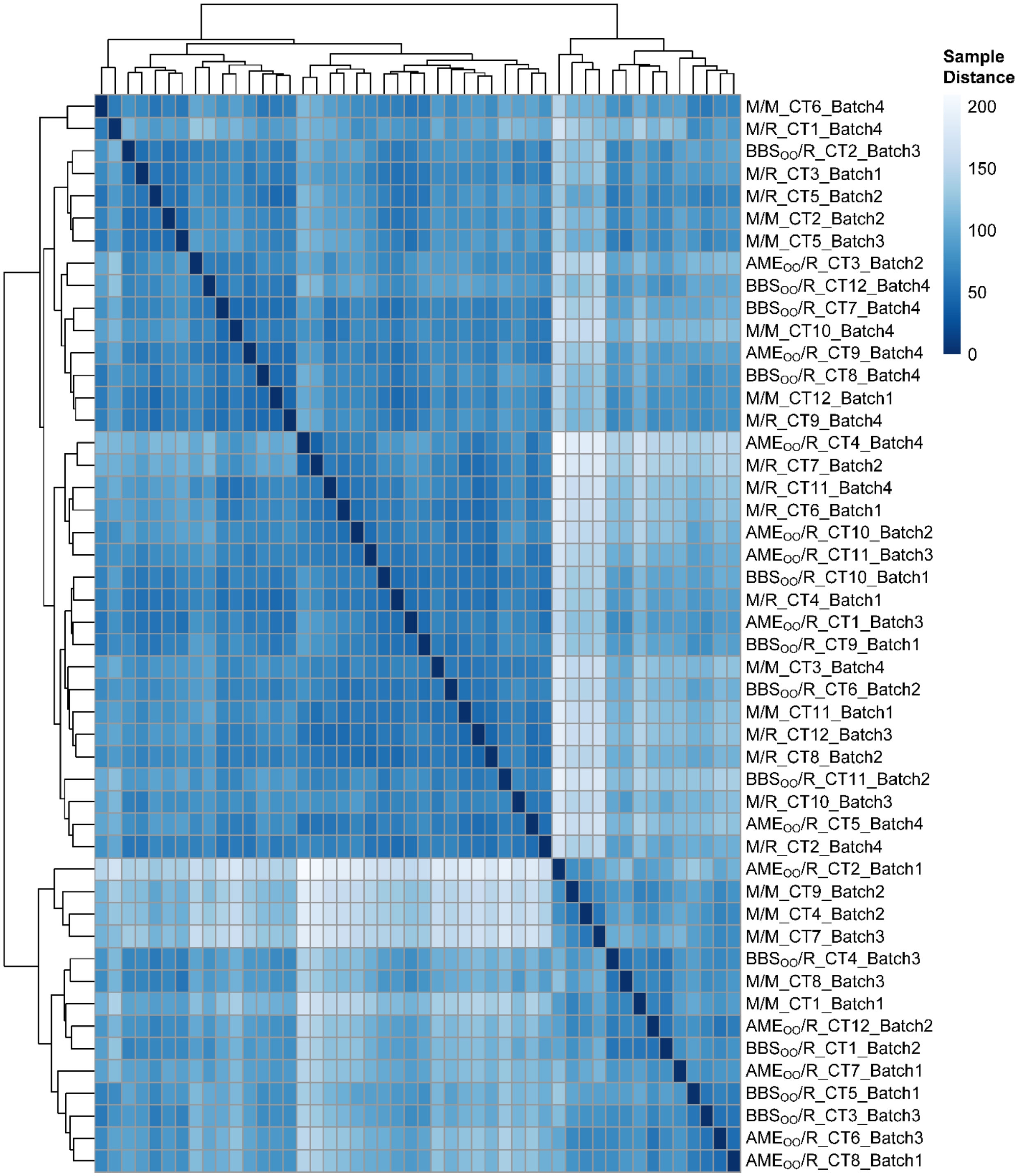

Supplementary Figure 3. Sample to sample distance matrix revealed differences between cecal tonsil transcriptomic datasets from 2 DPI with *S*. Reading. Euclidean distances were calculated using variance stabilized read counts for each sample. Samples are shown in the same order in the rows and columns; rows are labeled by vaccination/challenge group, tissue number (CT#), and isolation batch (1-4). M/M = mock-vaccinated/mock-challenged; M/R = mock-vaccinated/*S*. Reading-challenged; AME$_{OO}$/R = AviPro® Megan® Egg-vaccinated via oral gavage/*S*. Reading-challenged; BBS$_{OO}$/R = BBS 866-vaccinated via oral gavage/*S*. Reading-challenged; DPI = days post-inoculation.
